# Supplementary material for: Modeling glioblastoma heterogeneity as a dynamic network of cell states
Source: Mol Syst Biol. 2021 Sep 16;17(9):e10105. doi: 10.15252/msb.202010105 (PMC8444284; doi:10.15252/msb.202010105)
Supplement: Supplementary file 6 — Source Data for Figure 5 [file MSB-17-e10105-s004.zip › Figure5A_sourcedata/GSEA_3017/nosigdb_stateD.GseaPreranked.1622795010397/GAROFANO_IAVARONE_PPR_SUBTYPE.html]

Details for gene set GAROFANO\_IAVARONE\_PPR\_SUBTYPE[GSEA]

|  || Dataset | state23017 |
| Phenotype | NoPhenotypeAvailable |
| Upregulated in class | na\_neg |
| GeneSet | GAROFANO\_IAVARONE\_PPR\_SUBTYPE |
| Enrichment Score (ES) | -0.4576921 |
| Normalized Enrichment Score (NES) | -2.6631193 |
| Nominal p-value | 0.0 |
| FDR q-value | 0.0 |
| FWER p-Value | 0.0 |
Table: GSEA Results Summary

  

Fig 1: Enrichment plot: GAROFANO\_IAVARONE\_PPR\_SUBTYPE      
 Profile of the Running ES Score & Positions of GeneSet Members on the Rank Ordered List

  

| PROBE | GENE SYMBOL | GENE\_TITLE | RANK IN GENE LIST | RANK METRIC SCORE | RUNNING ES | CORE ENRICHMENT || 1 | SOX11 |  |  | 6 | 0.578 | 0.0016 | No |
| 2 | MEX3A |  |  | 30 | 0.402 | -0.0137 | No |
| 3 | PCDH10 |  |  | 68 | 0.318 | -0.0422 | No |
| 4 | SRRT |  |  | 74 | 0.314 | -0.0429 | No |
| 5 | HMGB3 |  |  | 80 | 0.311 | -0.0435 | No |
| 6 | TROAP |  |  | 109 | 0.283 | -0.0646 | No |
| 7 | ZNF22 |  |  | 119 | 0.276 | -0.0692 | No |
| 8 | LSM5 |  |  | 123 | 0.274 | -0.0686 | No |
| 9 | BIRC5 |  |  | 130 | 0.271 | -0.0706 | No |
| 10 | SEPHS1 |  |  | 146 | 0.260 | -0.0806 | No |
| 11 | TUBA1A |  |  | 148 | 0.259 | -0.0784 | No |
| 12 | NUP205 |  |  | 165 | -0.251 | -0.0894 | No |
| 13 | ZNF519 |  |  | 166 | -0.251 | -0.0865 | No |
| 14 | MKI67 |  |  | 206 | -0.254 | -0.1174 | No |
| 15 | DDX11 |  |  | 211 | -0.255 | -0.1179 | No |
| 16 | MCM2 |  |  | 226 | -0.256 | -0.1271 | No |
| 17 | PXMP2 |  |  | 233 | -0.257 | -0.1293 | No |
| 18 | SASS6 |  |  | 261 | -0.260 | -0.1498 | No |
| 19 | RBL1 |  |  | 285 | -0.262 | -0.1667 | No |
| 20 | FUS |  |  | 290 | -0.263 | -0.1671 | No |
| 21 | TRIP13 |  |  | 294 | -0.263 | -0.1666 | No |
| 22 | POLA2 |  |  | 312 | -0.264 | -0.1783 | No |
| 23 | LSM6 |  |  | 322 | -0.266 | -0.1830 | No |
| 24 | KNTC1 |  |  | 329 | -0.267 | -0.1851 | No |
| 25 | C18orf54 |  |  | 345 | -0.268 | -0.1950 | No |
| 26 | EXOSC8 |  |  | 347 | -0.268 | -0.1927 | No |
| 27 | CTDSPL2 |  |  | 358 | -0.269 | -0.1983 | No |
| 28 | C2orf80 |  |  | 374 | -0.271 | -0.2082 | No |
| 29 | RIF1 |  |  | 403 | -0.274 | -0.2293 | No |
| 30 | PNRC2 |  |  | 406 | -0.274 | -0.2278 | No |
| 31 | CCDC150 |  |  | 413 | -0.275 | -0.2298 | No |
| 32 | MASTL |  |  | 414 | -0.275 | -0.2266 | No |
| 33 | HAUS3 |  |  | 419 | -0.275 | -0.2268 | No |
| 34 | LCORL |  |  | 464 | -0.281 | -0.2619 | No |
| 35 | FOXM1 |  |  | 478 | -0.282 | -0.2699 | No |
| 36 | ECT2 |  |  | 481 | -0.283 | -0.2683 | No |
| 37 | MCM10 |  |  | 515 | -0.287 | -0.2936 | No |
| 38 | CENPP |  |  | 534 | -0.289 | -0.3059 | No |
| 39 | TRRAP |  |  | 537 | -0.289 | -0.3042 | No |
| 40 | GAB1 |  |  | 542 | -0.290 | -0.3043 | No |
| 41 | CENPO |  |  | 550 | -0.291 | -0.3070 | No |
| 42 | WDR76 |  |  | 557 | -0.292 | -0.3088 | No |
| 43 | DBF4B |  |  | 579 | -0.296 | -0.3236 | No |
| 44 | DSCC1 |  |  | 596 | -0.299 | -0.3340 | No |
| 45 | SPAG5 |  |  | 605 | -0.301 | -0.3374 | No |
| 46 | RFC2 |  |  | 607 | -0.302 | -0.3347 | No |
| 47 | PRC1 |  |  | 645 | -0.310 | -0.3633 | No |
| 48 | DNMT1 |  |  | 659 | -0.312 | -0.3710 | No |
| 49 | HAUS8 |  |  | 678 | -0.315 | -0.3829 | No |
| 50 | CHAF1A |  |  | 679 | -0.315 | -0.3792 | No |
| 51 | TMPO |  |  | 688 | -0.317 | -0.3825 | No |
| 52 | PLK4 |  |  | 699 | -0.319 | -0.3874 | No |
| 53 | KIF4A |  |  | 706 | -0.320 | -0.3889 | No |
| 54 | FIGNL1 |  |  | 717 | -0.322 | -0.3938 | No |
| 55 | NEIL3 |  |  | 720 | -0.323 | -0.3917 | No |
| 56 | SPC24 |  |  | 747 | -0.330 | -0.4105 | No |
| 57 | GINS1 |  |  | 780 | -0.338 | -0.4344 | No |
| 58 | MCM4 |  |  | 783 | -0.339 | -0.4321 | No |
| 59 | CDC25C |  |  | 792 | -0.341 | -0.4351 | No |
| 60 | PKMYT1 |  |  | 795 | -0.342 | -0.4328 | No |
| 61 | FANCI |  |  | 801 | -0.345 | -0.4331 | No |
| 62 | BRCA2 |  |  | 808 | -0.347 | -0.4342 | No |
| 63 | H2AFX |  |  | 810 | -0.347 | -0.4310 | No |
| 64 | BLM |  |  | 812 | -0.348 | -0.4278 | No |
| 65 | CENPM |  |  | 818 | -0.349 | -0.4280 | No |
| 66 | MAD2L1 |  |  | 819 | -0.350 | -0.4239 | No |
| 67 | CBX5 |  |  | 842 | -0.356 | -0.4389 | No |
| 68 | CENPJ |  |  | 849 | -0.357 | -0.4399 | No |
| 69 | RFC3 |  |  | 852 | -0.358 | -0.4374 | No |
| 70 | CDKN1B |  |  | 862 | -0.359 | -0.4410 | No |
| 71 | WDR62 |  |  | 881 | -0.366 | -0.4524 | No |
| 72 | SMC1A |  |  | 885 | -0.367 | -0.4507 | No |
| 73 | STIL |  |  | 888 | -0.369 | -0.4481 | No |
| 74 | CKAP2 |  |  | 900 | -0.375 | -0.4533 | Yes |
| 75 | TK1 |  |  | 902 | -0.375 | -0.4497 | Yes |
| 76 | SAP30 |  |  | 905 | -0.376 | -0.4470 | Yes |
| 77 | FANCA |  |  | 910 | -0.378 | -0.4461 | Yes |
| 78 | INCENP |  |  | 914 | -0.379 | -0.4442 | Yes |
| 79 | DEPDC1B |  |  | 923 | -0.383 | -0.4467 | Yes |
| 80 | DSN1 |  |  | 926 | -0.385 | -0.4439 | Yes |
| 81 | MSH2 |  |  | 927 | -0.385 | -0.4394 | Yes |
| 82 | KIF18B |  |  | 935 | -0.388 | -0.4409 | Yes |
| 83 | SKA1 |  |  | 950 | -0.395 | -0.4484 | Yes |
| 84 | DTL |  |  | 956 | -0.397 | -0.4481 | Yes |
| 85 | SKA3 |  |  | 965 | -0.399 | -0.4504 | Yes |
| 86 | HIRIP3 |  |  | 970 | -0.402 | -0.4491 | Yes |
| 87 | RTKN2 |  |  | 973 | -0.403 | -0.4461 | Yes |
| 88 | FANCD2 |  |  | 976 | -0.405 | -0.4431 | Yes |
| 89 | CEP78 |  |  | 984 | -0.408 | -0.4444 | Yes |
| 90 | CDC45 |  |  | 1000 | -0.413 | -0.4526 | Yes |
| 91 | POLD3 |  |  | 1002 | -0.415 | -0.4486 | Yes |
| 92 | KIF11 |  |  | 1003 | -0.416 | -0.4437 | Yes |
| 93 | CDC6 |  |  | 1008 | -0.418 | -0.4422 | Yes |
| 94 | MNS1 |  |  | 1009 | -0.419 | -0.4373 | Yes |
| 95 | TIMELESS |  |  | 1024 | -0.429 | -0.4444 | Yes |
| 96 | NCAPG2 |  |  | 1027 | -0.430 | -0.4411 | Yes |
| 97 | POLQ |  |  | 1033 | -0.436 | -0.4403 | Yes |
| 98 | TPX2 |  |  | 1034 | -0.437 | -0.4352 | Yes |
| 99 | LIG1 |  |  | 1035 | -0.437 | -0.4301 | Yes |
| 100 | CEP152 |  |  | 1040 | -0.439 | -0.4284 | Yes |
| 101 | FANCB |  |  | 1044 | -0.443 | -0.4258 | Yes |
| 102 | KIF22 |  |  | 1051 | -0.446 | -0.4257 | Yes |
| 103 | BRIP1 |  |  | 1052 | -0.447 | -0.4205 | Yes |
| 104 | SMC2 |  |  | 1062 | -0.452 | -0.4230 | Yes |
| 105 | EZH2 |  |  | 1070 | -0.455 | -0.4237 | Yes |
| 106 | PLK1 |  |  | 1082 | -0.460 | -0.4279 | Yes |
| 107 | MND1 |  |  | 1083 | -0.461 | -0.4225 | Yes |
| 108 | DPF1 |  |  | 1084 | -0.462 | -0.4170 | Yes |
| 109 | POLA1 |  |  | 1088 | -0.464 | -0.4142 | Yes |
| 110 | ATAD5 |  |  | 1091 | -0.465 | -0.4104 | Yes |
| 111 | ZWINT |  |  | 1099 | -0.467 | -0.4110 | Yes |
| 112 | BUB1B |  |  | 1111 | -0.477 | -0.4150 | Yes |
| 113 | RACGAP1 |  |  | 1112 | -0.479 | -0.4094 | Yes |
| 114 | E2F8 |  |  | 1116 | -0.485 | -0.4063 | Yes |
| 115 | BARD1 |  |  | 1117 | -0.486 | -0.4005 | Yes |
| 116 | NCAPD3 |  |  | 1130 | -0.496 | -0.4051 | Yes |
| 117 | ASF1B |  |  | 1133 | -0.498 | -0.4010 | Yes |
| 118 | PCNA |  |  | 1142 | -0.504 | -0.4021 | Yes |
| 119 | CKAP2L |  |  | 1147 | -0.508 | -0.3996 | Yes |
| 120 | FAM111B |  |  | 1151 | -0.514 | -0.3961 | Yes |
| 121 | NUSAP1 |  |  | 1154 | -0.515 | -0.3918 | Yes |
| 122 | GMNN |  |  | 1155 | -0.516 | -0.3857 | Yes |
| 123 | CEP135 |  |  | 1156 | -0.517 | -0.3796 | Yes |
| 124 | DBF4 |  |  | 1157 | -0.520 | -0.3735 | Yes |
| 125 | CCNF |  |  | 1160 | -0.522 | -0.3691 | Yes |
| 126 | RRM1 |  |  | 1166 | -0.534 | -0.3672 | Yes |
| 127 | KIF14 |  |  | 1168 | -0.535 | -0.3617 | Yes |
| 128 | BRCA1 |  |  | 1171 | -0.541 | -0.3571 | Yes |
| 129 | XRCC2 |  |  | 1180 | -0.551 | -0.3576 | Yes |
| 130 | KIF2C |  |  | 1183 | -0.558 | -0.3528 | Yes |
| 131 | ASPM |  |  | 1189 | -0.567 | -0.3504 | Yes |
| 132 | FEN1 |  |  | 1191 | -0.569 | -0.3446 | Yes |
| 133 | NUF2 |  |  | 1194 | -0.574 | -0.3396 | Yes |
| 134 | CKS1B |  |  | 1198 | -0.577 | -0.3354 | Yes |
| 135 | GTSE1 |  |  | 1210 | -0.602 | -0.3379 | Yes |
| 136 | CLSPN |  |  | 1212 | -0.605 | -0.3316 | Yes |
| 137 | KIF15 |  |  | 1213 | -0.610 | -0.3245 | Yes |
| 138 | CENPK |  |  | 1220 | -0.619 | -0.3224 | Yes |
| 139 | DUT |  |  | 1223 | -0.633 | -0.3167 | Yes |
| 140 | SMC4 |  |  | 1224 | -0.638 | -0.3092 | Yes |
| 141 | MYBL2 |  |  | 1225 | -0.639 | -0.3017 | Yes |
| 142 | NCAPH |  |  | 1228 | -0.643 | -0.2958 | Yes |
| 143 | KIF18A |  |  | 1231 | -0.649 | -0.2899 | Yes |
| 144 | CDCA8 |  |  | 1239 | -0.669 | -0.2881 | Yes |
| 145 | CENPA |  |  | 1243 | -0.679 | -0.2828 | Yes |
| 146 | CDCA5 |  |  | 1244 | -0.685 | -0.2747 | Yes |
| 147 | AURKB |  |  | 1250 | -0.700 | -0.2708 | Yes |
| 148 | MELK |  |  | 1251 | -0.701 | -0.2626 | Yes |
| 149 | CCNA2 |  |  | 1255 | -0.716 | -0.2567 | Yes |
| 150 | UBE2T |  |  | 1256 | -0.719 | -0.2483 | Yes |
| 151 | CDCA2 |  |  | 1258 | -0.727 | -0.2406 | Yes |
| 152 | ARHGAP11A |  |  | 1262 | -0.735 | -0.2345 | Yes |
| 153 | HMGB2 |  |  | 1263 | -0.736 | -0.2259 | Yes |
| 154 | RAD51AP1 |  |  | 1264 | -0.741 | -0.2171 | Yes |
| 155 | KIF23 |  |  | 1265 | -0.741 | -0.2084 | Yes |
| 156 | KIFC1 |  |  | 1267 | -0.748 | -0.2005 | Yes |
| 157 | TACC3 |  |  | 1268 | -0.751 | -0.1916 | Yes |
| 158 | TYMS |  |  | 1272 | -0.770 | -0.1852 | Yes |
| 159 | ATAD2 |  |  | 1275 | -0.775 | -0.1778 | Yes |
| 160 | NDC80 |  |  | 1278 | -0.810 | -0.1700 | Yes |
| 161 | ESCO2 |  |  | 1283 | -0.846 | -0.1635 | Yes |
| 162 | HIST2H2AC |  |  | 1288 | -0.880 | -0.1566 | Yes |
| 163 | HJURP |  |  | 1290 | -0.897 | -0.1469 | Yes |
| 164 | SPC25 |  |  | 1294 | -0.932 | -0.1386 | Yes |
| 165 | RRM2 |  |  | 1295 | -0.933 | -0.1276 | Yes |
| 166 | KPNA2 |  |  | 1297 | -0.972 | -0.1170 | Yes |
| 167 | PIF1 |  |  | 1299 | -0.987 | -0.1063 | Yes |
| 168 | NCAPG |  |  | 1303 | -1.015 | -0.0969 | Yes |
| 169 | CDK1 |  |  | 1311 | -1.433 | -0.0861 | Yes |
| 170 | UBE2C |  |  | 1312 | -1.466 | -0.0689 | Yes |
| 171 | TOP2A |  |  | 1313 | -1.528 | -0.0509 | Yes |
| 172 | HIST1H1B |  |  | 1318 | -1.922 | -0.0317 | Yes |
| 173 | HIST1H4C |  |  | 1320 | -2.769 | 0.0000 | Yes |
Table: GSEA details [plain text format]

  

Fig 2: GAROFANO\_IAVARONE\_PPR\_SUBTYPE: Random ES distribution      
 Gene set null distribution of ES for **GAROFANO\_IAVARONE\_PPR\_SUBTYPE**
